# Antiretroviral Therapy Use, Viral Detectability and Fear of Onward Transmission Among People Living with HIV in Australia: Changes Between 1997 and 2018

**DOI:** 10.1007/s10461-022-03795-2

**Published:** 2022-07-15

**Authors:** Thomas Norman, Adam Bourne, Anthony Lyons, John Rule, Jennifer Power

**Affiliations:** 1 Australian Research Centre in Sex, Health and Society, La Trobe University, Building NR6, Bundoora, VIC 3086 Australia; 2 Kirby Institute, UNSW Sydney, Sydney, Australia; 3 National Association of People with HIV Australia (NAPWHA), Newtown, Australia; 4 School of Population Health, University of NSW, Sydney, Australia

**Keywords:** HIV, Transmission, Antiretroviral therapy, Fear, Viral load

## Abstract

This paper examines how antiretroviral therapy (ART) use and fears towards the onward transmission of HIV have changed among people living with HIV (PLHIV) in Australia between 1997 and 2018. Participants were recruited as part of the HIV Futures study, a large cross-sectional survey of PLHIV in Australia, in 1997, 2003, 2012 and 2018 (total n = 3889). ART use, viral load detectability, and fear of onward HIV transmission were compared between years. Predictors of onward transmission fear were assessed among the 2018 subsample. While ART use within our sample decreased between 1997 and 2003, it subsequently increased to 97% in 2018. Self-reported viral load undetectability steadily increased over time, up to 88% in 2018. Notably, fewer PLHIV reported being fearful of transmitting HIV in 2018 compared to all other years. Being unfamiliar with the undetectable = untransmissible health movement, and having a detectable or uncertain viral load at last test, were significant predictors of being fearful of onward HIV transmission. Beyond the immediate medical considerations of HIV treatment, these results suggest that the undetectable = untransmissible movement may play a critical role in attenuating burdens experienced by PLHIV in Australia and that such messaging, in tandem with early and consistent ART use, should remain a salient feature of heath messaging among this population.

## Introduction

One of the primary benefits of modern antiretroviral therapy (ART) and subsequent viral load suppression is the eliminated risk of onward transmission of HIV through sexual contact. In 2016, the largest study of transmission among HIV serodiscordant partners demonstrated that participants who started ART soon after diagnosis saw a 93% reduction in onward transmission, while zero individuals who were virally supressed transmitted HIV to their negative partner [1]. Although this sample predominantly featured heterosexual couples, such findings were similarly demonstrated among gay and bisexual men who have sex with men within the following 2 years [2, 3]. This spurred the “undetectable = untransmissible” (U = U) movement from 2016 onward; major health and community messaging (e.g., through direct discussion with clinicians or via social marketing campaigns run by community organisations) indicating that individuals with an undetectable viral load were unable to transmit HIV sexually. While a watershed moment in HIV treatment and prevention, the benefits of U = U also tied into the social and mental well-being of people living with HIV (PLHIV). For the first time since the emergence of HIV, there was robust clinical evidence that onward transmission could be eliminated with consistent ART adherence. For PLHIV, the U = U message potentially reduces stigma and anxiety associated with sexual transmission of HIV. This may support PLHIV to initiate conversations about HIV with prospective sexual partners and increase confidence to pursue dating and sexual relationships among people who have struggled with this due to HIV-related stigma [4, 5].

Fear of transmitting HIV to sexual partners has been a major concern for PLHIV and has significantly diminished their access to satisfying and pleasurable sex. For example, among a sample of gay and bisexual men living with HIV in the United Kingdom (n = 1217), over 70% reported one or more problems with sex in the previous 12 months, including loss of libido, poor self-image or loss of confidence, worries of onward HIV transmission and fears of rejection from sexual partners [6]. Unfortunately, such problems are often at odds with what PLHIV aspire to in their sex lives, including desire for sex which is emotionally or psychologically connected, and free from physical, social or psychological harm [7]. Caring for the emotional and sexual needs of PLHIV is critical in improving the broader well-being of this population, particularly in current policy contexts where the increasing medicalisation of HIV care and prevention has been prioritised [8]. In this sense, it is important to identify whether fears surrounding onward transmission have reduced since the era of U = U messaging, and whether awareness of U = U is a factor for lower levels of fear.

U = U messaging has been the focus of major international and local advocacy campaigns and has been incorporated into Australian clinical guidelines regarding HIV prevention to encourage clinicians to endorse and promote the message with their patients [9]. A multi-country study in 2020 found that approximately 66% of PLHIV had discussed U = U with their health care provider [4]. In Australia, despite the proliferation of U = U messaging, a cross-sectional survey conducted in 2018 by Huntingdon, de Wit [10] noted that while 70.5% of their sample of PLHIV believed in the concept of treatment as a prevention strategy, only 48.2% were confident in it as an effective prevention strategy for onward transmission of HIV in sexual situations. However, U = U is part of a longer history of ART use in Australia and it is likely that 48% of PLHIV reporting confidence in use of ART as a prevention technology represents a significant change in the way PLHIV view ART relative to the pre-U = U period. Indeed, a multi-year study among exclusively gay and bisexual men in Australia demonstrated that belief in treatment as prevention markedly increased between 2013 and 2019—particularly among HIV positive men [11]. Qualitative work has similarly explored the reimagining of sexuality in the wake of treatment as prevention, with Persson [12] highlighting its potential to reframe sexual relationships among serodiscordant couples as both safe and legitimate, and lessening fears of onward transmission.

While previous studies have investigated willingness for and comfort in relying on viral suppression as a prevention strategy [12–14], the ways in which explicit fear of onward transmission of HIV has changed among PLHIV in Australia since the introduction of modern ART, and subsequent increased awareness about U = U has not been formally investigated. This therefore calls for greater insight into how such attitudes have changed alongside ART use and ART efficacy, the impact of U = U messaging, and what more may need to be done. Moreover, in addition to analysing changes in attitudes over time, identifying relevant predictors of concerns about onward transmission among PLHIV may identify gaps in U = U messaging with respect to sub-populations or treatment, health or life experiences of PLHIV.

This paper examines how ART use, viral detectability and attitudes towards the onward transmission of HIV have changed among PLHIV in Australia between 1997 and 2018. The primary aims of this paper are to (i) establish how ART use and viral load detectability has changed since 1997, (ii) determine changes in fear about the risk of onward HIV transmission over this period, and (iii) establish the contemporary relationship between demographic and treatment characteristics of PLHIV and their concerns towards onward HIV transmission.

## Methods

HIV Futures is a periodic, cross-sectional survey of PLHIV in Australia that was established in 1997. Data for this paper were extracted from four iterations of the HIV Futures study: HIV Futures 1 (conducted in 1997), HIV Futures 4 (conducted in 2003), HIV Futures 7 (conducted 2012) and HIV Futures 9 (conducted in late 2018, after the publication of the ‘Opposites Attract’ study demonstrating U = U among gay and bisexual men who have sex with men [2]. A new sample of participants was recruited for each iteration, albeit using similar methods of recruitment. Participants completed pen-and-paper or online (commencing from HIV Futures 5 in 2006) surveys. Surveys were distributed via community HIV health organisations and clinic waiting rooms, while the online survey was promoted via targeted paid advertising in relevant magazines and social media, such as Facebook and Instagram, and on dating/sexual networking applications utilised by gay and bisexual men. Full study details have been published elsewhere [15].

A total of 3,889 survey responses were received across all study iterations reported on in this paper: 925 in HIV Futures 1 (23.8%), 1058 in HIV Futures 4 (27.3%), 1059 in HIV Futures 7 (27.2%), and 847 in HIV Futures 9 (21.8%).

### Key Measures

The HIV Futures questionnaire includes a range of items related to health and wellbeing, finances and employment, antiretroviral use, HIV diagnosis and testing, drug use, sex and relationships. For every iteration of the survey, participants were asked to report a range of demographic information (e.g., age, sex, gender, level of education), whether they were currently on ART (yes/no), and the result of their last viral load test (detectable/undetectable/I don’t know). They were also asked to report their attitudes towards fear of onward HIV transmission (‘I am afraid of infecting my partner, or potential partner, with HIV’), with responses on a 5-point Likert scale (strongly disagree – strongly agree). This item was derived by the original HIV Futures research team and has remained consistent throughout every iteration. Participants in HIV Futures 9 were asked whether they were familiar with U = U messaging (“Are you familiar with the U = U [undetectable = untransmissible] campaign?”; familiar/unfamiliar/don’t know), and the extent to which they experienced HIV-related stigma in the last 12 months (“In the last 12-months, to what extent have you experienced any stigma or discrimination [e.g. avoidance, pity, blame, shame, rejection, verbal abuse, bullying] in relation to your HIV status: never/rarely/sometimes/often/always).

Ethics approval for the study was granted through the La Trobe University Human Ethics Committee (S15-100).

### Data Analysis

All analyses were conducted in IBM SPSS version 28.

Sample characteristics were computed for the whole sample (n = 3889). Fear of onward transmission responses were dichotomised into ‘agree’ and ‘not agree’ (i.e., ‘strongly agree’ and ‘agree’ were collapsed into ‘agree’, while ‘strongly disagree’ and ‘disagree’ were collapsed into ‘not agree’). As this analysis focuses on participants who categorically agree they are afraid of transmitting HIV to a partner, non-committal responses (e.g., ‘I neither agree nor disagree’) were also collapsed into the ‘not agree’ level (total responses, n = 3638). Latest viral load test responses were dichotomised into ‘undetectable’ and ‘detectable/don’t know’ (total responses, n = 3497). Unknown and detectable viral load were combined as we cannot assume an unknown viral load is undetectable and therefore, when it comes to concerns about onward transmission, should be managed as if it were detectable. Experience of HIV-related stigma in the past 12 months was dichotomised into ‘rarely/never’ and ‘sometimes/often/always’.

Chi-square tests were used to compare differences in ART use, result of last viral load test and fear of onward transmission between all study years (i.e., 1997, 2003, 2012 and 2018). Where familywise differences were observed, bivariate differences were calculated.

A multivariable logistic regression was then performed using the HIV Futures 9 subsample (2018) to determine the relationship between the primary demographic and treatment characteristics asked about in the survey and fear of onward HIV transmission, using the following binary predictors: age (< 35 years vs 35 + years), gender (male vs female), sexuality (gay/bisexual/a different sexuality vs heterosexual), residential location (city/suburban vs regional/rural), level of education (university degree vs no university degree), ever heard of U = U (familiar vs unfamiliar/don’t know), income source [salary vs no salary (e.g., pension, superannuation, family support)], viral load at last test (undetectable vs detectable/don’t know), time living with HIV (< 10 years vs 10 + years) and self-reported health (poor/fair vs good/excellent). For this section of analysis a multiple imputation approach was used, with 5 imputations, to address missing data across the covariates [16]. Regression results are based on the resulting pooled dataset (n = 820). Adjusted odds ratios, confidence intervals and *p* values are reported.

## Results

### Sample Characteristics

Sample characteristics are demonstrated in Table 1. The total sample comprised 3,889 participants, with a mean age of 45. The majority of the sample (90.3%) were male, identified as gay (77.9%) and lived in capital cities or inner suburban areas (61%).

**Table 1:** Sample characteristics

	1997 (N = 925)	2003 (N = 1059)	2012 (N = 1058)	2018 (N = 847)	Total sample (N = 3889)
Age (M, Range)	39.3 (18–77)	44.1 (19–92)	49 (19–84)	49.8 (18–85)	45.5 (18–92)
Gender (%)					
Male	90.8	90.7	92.9	86.2	90.3
Female	9.2	9.1	6.7	9.6	8.6
Transgender/gender diverse	0	0.3	0.4	4.2	1.1
Sexuality (%)					
Gay/homosexual/lesbian	75.5	77.6	83.3	74.1	77.9
Straight/heterosexual	15.7	14.9	10.4	14.9	13.9
Bisexual	8.3	5.8	4.5	5.4	6
A different sexuality	0.4	1.6	1.8	5.6	2.2
Residential location (%)					
Capital city/inner suburban	62.1	60.3	59.4	62.4	61
Outer suburban	17.7	12.5	14.9	15.7	15.1
Regional centre	12.5	17	16	14.9	15.2
Rural	7.7	10.2	9.7	7	8.8
Education (%)					
University degree	25.7	26.2	40.7	45.5	34
No university degree	74.3	73.8	59.3	54.5	66
Income source (%)					
Salary	34	37.1	46.9	52	42.3
No salary	66	62.9	53.1	48	57.7
Self-reported health (%)					
Poor/fair	32.9	32.9	28.8	49.5	35.3
Good/excellent	67.1	67.1	71.2	50.5	64.7
Experience of HIV-related stigma or discrimination (past 12 months)					
Never/rarely	N/A	N/A	N/A	67.9	N/A
Sometimes/often/always	N/A	N/A	N/A	32.1	N/A
Years since diagnosis (M, Range)	7.5 (0–18)	10.9 (0–23)	13.9 (0–31)	17 (0–37)	12 (0–37)
Heard of U = U (%)	N/A	N/A	N/A	74.8	N/A

U = U, undetectable equals untransmissible. Note: Some individuals who identify as transgender/gender diverse may have identified as ‘male’ or ‘female’

### ART Use

**Fig. 1:**
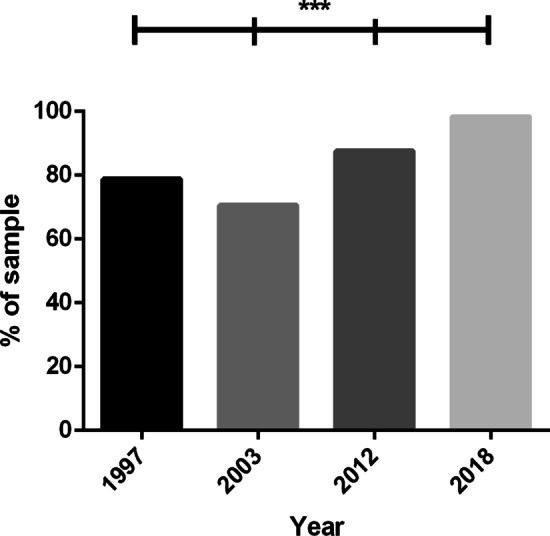
Proportion of sample who are currently taking ART, by year (n = 3889). ***Chi-square tests significant at the *p* < 0.001 level between all study years

Current ART use across study years is displayed in Fig. 1. A chi-square test indicated a difference between the proportion of participants who reported being on ART between study years [χ^2^(3, *N* = 3889) = 275.56, *p* < 0.001]. Participants in 2003 were significantly less likely to be using ART than in all other years {1997 [χ^2^(1, *N* = 1984) = 17.46, *p* < 0.001]; 2012 [χ^2^(1, *N* = 2117) = 91.67, *p* < 0.001]; 2018 [χ^2^(1, *N* = 1906) = 242.91, *p* < 0.001]}, participants in 1997 were significantly less likely to be taking ART than those in 2012 [χ^2^(1, *N* = 1983) = 26.90, *p* < 0.001] and 2018 [χ^2^(1, *N* = 1772) = 152.55, *p* < 0.001], and participants in 2012 were significantly less likely to be taking ART than those in 2018 [χ^2^(1, *N* = 1905) = 73.47, *p* < 0.001].

### Latest Viral Load Test

**Fig. 2:**
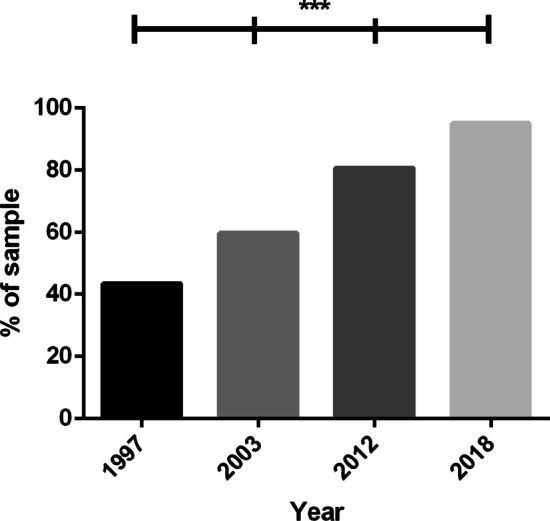
Proportion of sample who had an undetectable viral load at last test, by year (n = 3497). ***Chi-square tests significant at the *p* < 0.001 level between all study years

Undetectable viral load at last test, across study years, is displayed in Fig. 2. A chi-square test indicated a familywise difference in the proportion of individuals who reported an undetectable viral load between study years [χ^2^(3, *N* = 3497) = 442.19, *p* < 0.001]. Participants in 2018 were significantly more likely to have an undetectable viral load than all other years {1997 [χ^2^(1, *N* = 1608) = 358.01, *p* < 0.001]; 2003 [χ^2^(1, *N* = 1679) = 172.57, *p* < 0.001]; 2012 [χ^2^(1, *N* = 1795) = 34.70, *p* < 0.001]}, participants in 2012 were significantly more likely to have an undetectable viral load than 2003 [χ^2^(1, *N* = 1889) = 68.60, *p* < 0.001] and 1997 [χ^2^(1, *N* = 1819) = 223.71, *p* < 0.001], and participants in 2003 were significantly more likely to have an undetectable viral load than those in 1997 [χ^2^(1, *N* = 1702) = 45.98, *p* < 0.001].

### Fear of Onward HIV Transmission

**Fig. 3:**
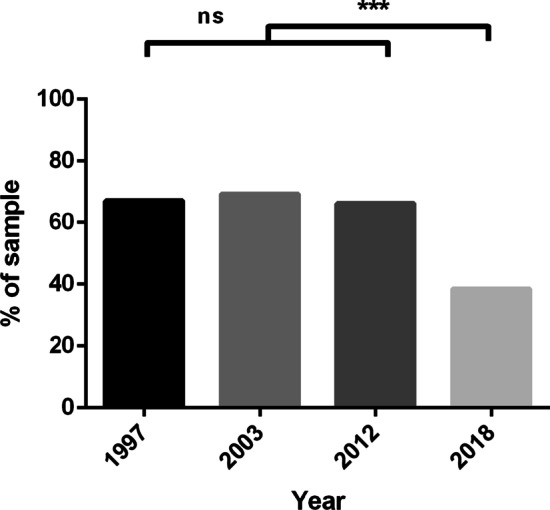
Proportion of sample who agree they are afraid of transmitting HIV to a partner or potential partner, by year (n = 3638). ***Chi-square tests significant at the *p* < 0.001 level between 2018 and 1997, 2003 and 2012. *ns* non-significant between 1997, 2003 and 2012

Fear of onward HIV transmission across study years is displayed in Fig. 3. A chi-square test indicated a difference in the proportion of participants between study years who agreed that they were fearful of onward transmission [χ^2^(3, *N* = 3638) = 208.15, *p* < 0.001]. Participants in 2018 were significantly less likely to agree that they were afraid of transmitting HIV than participants in 1997 [χ^2^(1, *N* = 1607) = 130.47, *p* < 0.001], 2003 [χ^2^(1, *N* = 1729) = 162.35, *p* < 0.001] and 2012 [χ^2^(1, *N* = 1730) = 131.08, *p* < 0.001]. Differences in fears of onward transmission between 1997, 2003 and 2012 were non-significant.

### Predictors of Fear of Onward HIV Transmission Among 2018 Sample

A multivariable logistic regression, with predictors of age, gender, sexuality, residential location, level of education, income source, experience of HIV-related stigma in past 12 months, self-reported health, awareness of U = U, viral load at last test, and time living with HIV, indicated improved model fit compared to the null [χ^2^(11, N = 820) = 25.25, *p* < 0.001]. Adjusted odds ratios and 95% CIs are displayed in Table 2. Participants who were unfamiliar with the concept of U = U, or selected don’t know, were 2.4 times more likely to ‘agree’ that they were afraid of transmitting HIV compared to those who were familiar with U = U (*p* < 0.001). Participants who reported detectable viral load, or were not sure of their status, were 2.6 times more likely to ‘agree’ that they were afraid of transmitting HIV compared to those who reported an undetectable viral load at last test (*p* = 0.002). All other predictors were non-significant.

**Table 2:** Logistic regression results for ‘I am afraid of infecting my partner, or potential partner, with HIV’ among the 2018 subsample (n = 820)

	‘I am afraid of infecting my partner, or potential partner, with HIV’			
	% of subsample that ‘agree’	aOR	95% CI	*p*-value
Age				
< 35	39	–	–	–
35 +	38	0.91	0.54–1.52	0.705
Gender				
Male	38	–	–	–
Female	38	0.78	0.37–1.67	0.529
Sexuality				
Homosexual/bisexual/a different sexuality	37	–	–	–
Heterosexual	39	1.11	0.58–2.13	0.741
Area of living				
Capital city/suburban	38	–	–	–
Regional/rural	38	1.10	0.76–1.60	0.621
Education				
University degree	42	–	–	–
No university degree	37	0.84	0.61–1.16	0.282
Income				
Salary	38	–	–	–
No salary	39	1.10	0.80–1.50	0.665
Self-reported health				
Poor/fair	39	–	–	–
Good/excellent	38	0.95	0.68–1.32	0.742
Experience of HIV-related stigma or discrimination (past 12 months)				
Never/rarely	39	–	–	–
Sometimes/often/always	38	0.94	0.63–1.41	0.758
Heard of U = U				
Familiar	34	–	–	–
Unfamiliar/don’t know	54	2.40	1.55–3.52	** < 0.001**
Viral load at last test				
Undetectable	36	–	–	–
Detectable/don’t know	62	2.58	1.55–4.27	** < 0.001**
Time living with HIV				
< 10 years	42	–	–	–
10 years +	38	0.86	0.55–1.35	0.498

‘–’ denotes reference categories. U = U, undetectable equals untransmissible

*CI* confidence interval, *aOR* adjusted odds ratio

## Discussion

This study identified notable changes in ART use, viral load at last test, and concerns towards onward HIV transmission among PLHIV in Australia between 1997 and 2018. It further identified two important predictors of fear of onward HIV transmission among the 2018 subsample: awareness of U = U (‘familiar’ vs ‘unfamiliar’), and viral load status at latest test (‘undetectable’ vs ‘detectable’/‘don’t know’).

Our findings indicate that ART use steadily increased between 1997 and 2018, with the exception of a decrease between 1997 and 2003. The introduction of the drug Indinavir in 1996 changed the landscape of HIV treatment globally, signifying the era of highly active antiretroviral therapy (HAART) [17]. Indinavir was used in combination with two other pre-existing drugs, a treatment strategy known as ‘triple combination therapy’. Combination therapy using Indinavir was markedly more effective than previous treatment options in both the suppression of HIV and in reducing disease-associated morbidity and mortality [17]. However, the side effect profile and treatment schedule of early HAART proved to be a barrier for many; it was a common for PLHIV to take treatment breaks to avoid side effects for intermittent periods [18]. The decrease in ART use between the introduction of HAART (in 1996) and 2003 likely reflects such practices. However, ART technologies soon advanced, producing less side effects and improving ART adherence. Thus, the proportion of individuals taking ART rose to 88% in 2012, and exceeded 97% in our 2018 subsample.

Despite a decrease in ART use in 2003, the proportion of participants reporting an undetectable viral load increased sequentially across study years, with 88% of participants in 2018 reporting an undetectable viral load as of their most recent test. While viral load undetectability steadily increased since 1997, the proportion of the sample who agreed they were afraid of transmitting HIV did not significantly differ between 1997, 2003 and 2012. However, importantly, participants were markedly less likely to report being afraid of transmitting HIV in 2018 compared to all other years. This is a critical finding, suggesting that participants in a post U = U climate were significantly less fearful of onward HIV transmission than participants in previous years; sitting in line with a shift in attitudes towards treatment as a means of prevention since 2012 [11], and even among a substantial proportion of HIV-positive men in casual sexual contexts [19].

Further to this, being unfamiliar with U = U and having a detectable (or being uncertain of their) viral load were significant predictors of being fearful of onward HIV transmission. Augmenting demonstrated links between U = U awareness in healthcare contexts and positive health outcomes such as treatment adherence and HIV disclosure [4], the findings of this study highlight the potential importance of both U = U and viral load undetectability for PLHIV in attenuating personal fears of onward transmission. Fear relating to the possibility of HIV transmission, as well as HIV related stigma from sexual partners, has historically significantly diminished the ability of PLHIV to access pleasurable sex [6]. The value of U = U in reshaping the sexual landscape for HIV positive people is becoming clear; not just in terms of direct clinical outcomes, but also in ameliorating concerns about onward transmission, as well as the social and sexual implications that follow. ‘Undetectability’ as a concept has begun to shift perceptions of risk and safety in serodiscordant sexual encounters and for serodiscordant couples [20, 21].

While our findings highlight encouraging trends, it is also important to note that a substantial minority of participants (38.5%) still reported being fearful of onward transmission. Although recent experiences of stigma were not predictive of fear of onward transmission among our sample, there remains a complex historical context that is attached to living with HIV; U = U is a relatively new 'phase' in the HIV response, and further reducing fears of onward transmission may be a slow process. It is thus imperative to continue monitoring trends in onward transmission fears and resource U = U promotion initiatives. Despite an increase in U = U messaging, it may be difficult for some PLHIV to navigate this despite viral undetectability, and further to this, some PLHIV will not be able to achieve undetectability, or struggle with treatment adherence. Viral load is now part of the negotiation of safe sex, along with pre-exposure prophylaxis (PrEP), and this may be challenging for people who struggle to achieve a consistently undetectable viral load. Ongoing efforts should centre on supporting these individuals, specifically addressing their needs, investigating how their intimacy and relationships are affected, and how they are managing in negotiating their sex lives.

The impact that HAART and viral undetectability has made on the lives of PLHIV has made important leaps since 1997. Mortality and morbidity associated with HIV infection has dramatically decreased; PLHIV are living longer, and spending more of those years with a good quality of life [22, 23]. However, ART use is only one dimension of living with HIV. Health responses to HIV must continue to extend beyond the immediate medical outcomes and include consideration of the broader well-being of PLHIV, including a constellation of factors such as attitudes, social wellbeing, relationships and minimising the negative impact of stigma. As evidenced by the findings of this study, U = U campaigning and viral load undetectability are associated with allaying fears of onward transmission of HIV in Australia and should thus remain a critical feature in the ongoing heath messaging for PLHIV.

### Strengths and Limitations

There are marked strengths of this study: it includes a large sample of PLHIV in Australia and is, to our knowledge, the first study in Australia to compare ART use, viral load detectability and fear of onward transmission over a 20 + year period. It also identified two important predictors of a fear of onward transmission: being unfamiliar with U = U messaging and having a detectable (or uncertain) viral load at last test.

However, it is important to note that this study only investigated changes in PLHIV who explicitly ‘agreed’ that they were afraid of transmitting HIV to a partner or potential partner. As non-committal responses (e.g., those who neither agreed nor disagreed they were afraid of transmitting HIV) were combined with those who ‘disagreed’, there may be distinctions between different levels of attitudes that were not identified in our analysis. In future studies, it may be useful to examine different degrees of fear of onward transmission among PLHIV (e.g., very, moderately, slightly), or further investigate specific concerns or understandings that participants have about the likelihood of transmission. Qualitative research might be particularly useful in exploring different ways in which PLHIV experience fears.

Further to this limitation, our analysis was based on cross-sectional data. Therefore, causality between the predictors used in our analysis and attitudes towards onward transmission cannot be inferred. Specifically, U = U knowledge and viral (un)detectability are simply associated with onward transmission attitudes and are not necessarily a direct result of changes. It is recommended that future research more explicitly investigates the relationship between U = U and onward transmission attitudes, beyond simple familiarity with U = U.

Finally, it should be noted that there is likely to be an underrepresentation of people who do not speak English as their first language in the HIV Futures sample. This is a difficult limitation to overcome given the diversity of Australia’s migrant population. The many languages spoken among people living with HIV who are born overseas means that meaningful translation of the survey instrument into all relevant languages, and the sensitive and long-term community engagement with diverse local communities to support survey recruitment, is beyond the resources currently available to the study. It is important to consider that individuals from broader cultural backgrounds, with varying experiences of stigma, migration experience and access to treatment, may not be adequately represented within our sample. Future work should endeavour to address these limitations through mixed methods studies and building diverse community partnerships.

## Conclusions

Our findings indicate that ART use and viral load undetectability amongst PLHIV in Australia have both markedly increased since 1997, while fears of onward transmission of HIV decreased in 2018 (compared to 1997, 2003 and 2012). Among our sample of PLHIV in 2018, being unfamiliar with U = U and having a detectable (or being uncertain of their) viral load were both significant predictors of being fearful of onward transmission. Beyond the immediate medical considerations of HIV treatment, these results suggest that U = U campaigns may play a critical role in attenuating burdens experienced by PLHIV and that such messaging, in tandem with early and consistent ART use, should remain a salient feature of health messaging among this population.

## Data Availability

Data available on request from authors.

## References

[1] Cohen MS, Chen YQ, McCauley M, Gamble T, Hosseinipour MC, Kumarasamy N (2016). Antiretroviral therapy for the prevention of HIV-1 transmission. N Engl J Med.

[2] Bavinton BR, Pinto AN, Phanuphak N, Grinsztejn B, Prestage GP, Zablotska-Manos IB (2018). Viral suppression and HIV transmission in serodiscordant male couples: an international, prospective, observational, cohort study. Lancet HIV.

[3] Rodger AJ, Cambiano V, Bruun T, Vernazza P, Collins S, Degen O (2019). Risk of HIV transmission through condomless sex in serodifferent gay couples with the HIV-positive partner taking suppressive antiretroviral therapy (PARTNER): final results of a multicentre, prospective, observational study. Lancet.

[4] Okoli C, Van de Velde N, Richman B, Allan B, Castellanos E, Young B (2021). Undetectable equals untransmittable (U = U): awareness and associations with health outcomes among people living with HIV in 25 countries. Sex Transm Infect.

[5] Rendina HJ, Talan AJ, Cienfuegos-Szalay J, Carter JA, Shalhav O (2020). Treatment is more than prevention: perceived personal and social benefits of undetectable = untransmittable messaging among sexual minority men living with HIV. AIDS Patient Care STDS.

[6] Bourne A, Hickson F, Keogh P, Reid D, Weatherburn P (2012). Problems with sex among gay and bisexual men with diagnosed HIV in the United Kingdom. BMC Public Health.

[7] Bourne A, Hammond G, Hickson F, Reid D, Schmidt AJ, Weatherburn P (2013). What constitutes the best sex life for gay and bisexual men? Implications for HIV prevention. BMC Public Health.

[8] Young I, Davis M, Flowers P, McDaid LM (2019). Navigating HIV citizenship: identities, risks and biological citizenship in the treatment as prevention era. Health Risk Soc.

[9] ASHM. Guidelines for health care providers for providing advice regarding U=U (undetectable = untransmissible). Australasian Society for HIV, Sexual Health and Viral Hepatitis Medicine (ASHM). 2020.

[10] Huntingdon B, de Wit J, Duracinsky M, Juraskova I (2020). Belief, covariates, and impact of the “undetectable = untransmittable” message among people living with HIV in Australia. AIDS Patient Care STDS.

[11] Holt M, MacGibbon J, Bear B, Lea T, Kolstee J, Crawford D (2021). Trends in belief that HIV treatment prevents transmission among gay and bisexual men in Australia: results of National Online Surveys 2013–2019. AIDS Educ Prev.

[12] Persson A (2016). 'The world has changed': pharmaceutical citizenship and the reimagining of serodiscordant sexuality among couples with mixed HIV status in Australia. Sociol Health Illn.

[13] Bavinton BR, Holt M, Grulich AE, Brown G, Zablotska IB, Prestage GP (2016). Willingness to act upon beliefs about ‘treatment as prevention’ among Australian gay and bisexual men. PLoS ONE.

[14] Holt M, Draper BL, Pedrana AE, Wilkinson AL, Stoové M (2018). Comfort relying on HIV pre-exposure prophylaxis and treatment as prevention for condomless sex: results of an online survey of australian gay and bisexual men. AIDS Behav.

[15] Power J, Brown G, Lyons A, Thorpe R, Dowsett GW, Lucke J (2017). HIV futures 8: protocol for a repeated cross-sectional and longitudinal survey of people living with HIV in Australia. Front Public Health.

[16] Jakobsen JC, Gluud C, Wetterslev J, Winkel P (2017). When and how should multiple imputation be used for handling missing data in randomised clinical trials—a practical guide with flowcharts. BMC Med Res Methodol.

[17] Vella S, Schwartländer B, Sow SP, Eholie SP, Murphy RL (2012). The history of antiretroviral therapy and of its implementation in resource-limited areas of the world. AIDS.

[18] Josefson D (2003). Breaks from antiretroviral treatment are not the answer for HIV patients. BMJ.

[19] Holt M, Lea T, Mao L, Zablotska I, Prestage G, de Wit J (2015). Brief report: HIV prevention by Australian gay and bisexual men with casual partners: the emergence of undetectable viral load as one of a range of risk reduction strategies. J Acquir Immune Defic Syndr.

[20] Persson A, Newman CE, Ellard J (2017). Breaking binaries? Biomedicine and serostatus borderlands among couples with mixed HIV status. Med Anthropol.

[21] Rule J, Slavin S, Persson A, Hughes SD (2017). Seeking seroharmony: changing conceptualisations of serodifference and serostatus. Cross-cultural perspectives on couples with mixed HIV status: beyond positive/negative.

[22] Oguntibeju OO (2012). Quality of life of people living with HIV and AIDS and antiretroviral therapy. HIV AIDS (Auckl).

[23] Wandeler G, Johnson LF, Egger M (2016). Trends in life expectancy of HIV-positive adults on antiretroviral therapy across the globe: comparisons with general population. Curr Opin HIV AIDS.

